# Supplementary material for: Persistent DNA-break potential near telomeres increases initiation of meiotic recombination on short chromosomes
Source: Nat Commun. 2019 Feb 27;10:970. doi: 10.1038/s41467-019-08875-x (PMC6393486; doi:10.1038/s41467-019-08875-x)
Supplement: Supplementary file 1 — Supplementary Information [file 41467_2019_8875_MOESM1_ESM.pdf]

Supplementary Information

Persistent DNA-break potential near telomeres increases initiation of meiotic recombination on short chromosomes

Subramanian et al.

This supplementary information file contains:

Supplementary Figures 1 to 8

Supplementary Tables 1 to 6

Supplementary References

Supplementary Figures

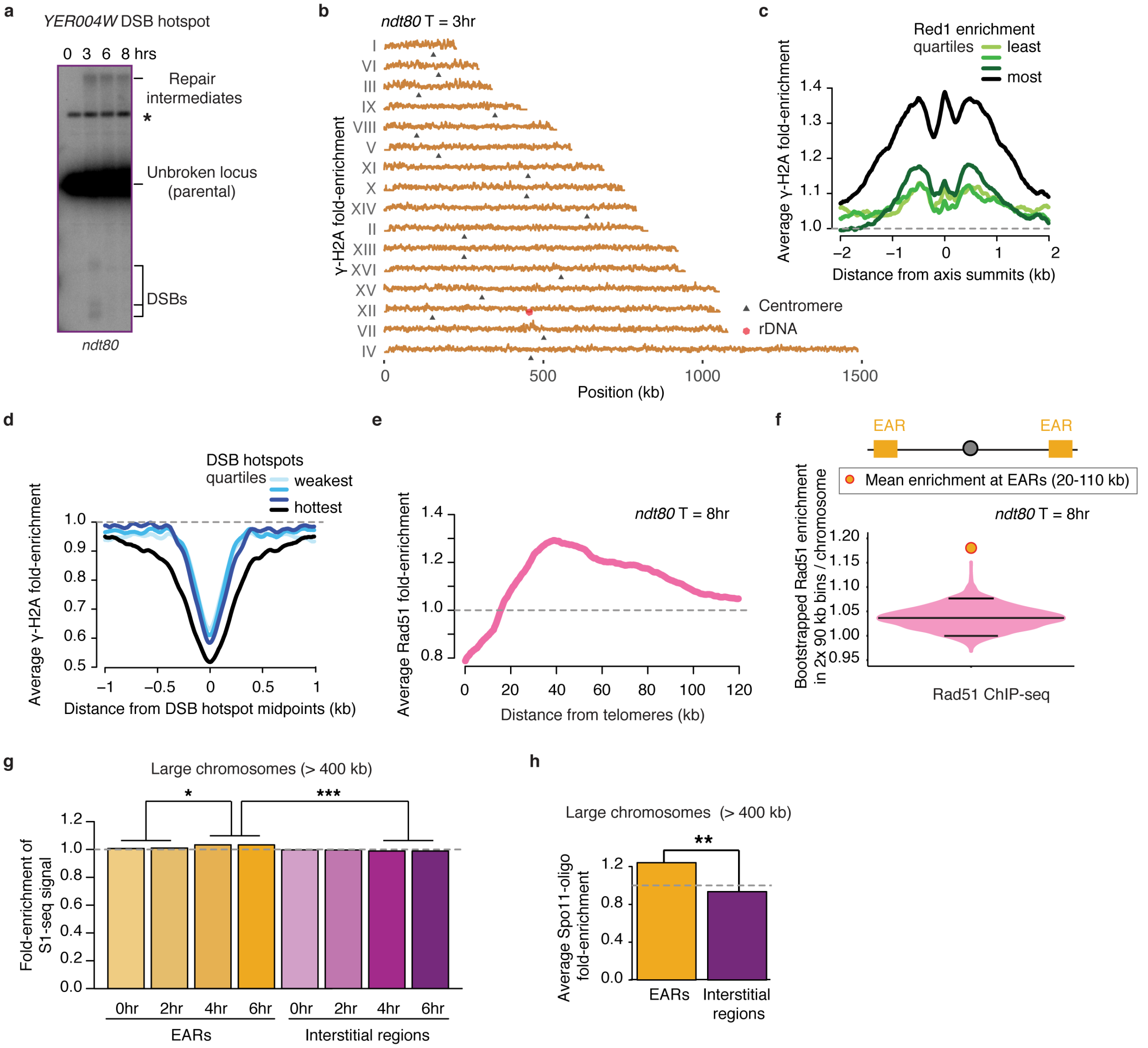

Supplementary Figure 1. Long-lived DSB hotspots are enriched in EARs.

(a) Southern analysis of DSBs from *ndt80Δ* cells progressing synchronously though meiotic prophase at *YER004W* (short-lived DSB hotspot, magenta outline). Unbroken parental fragments, DSBs and repair intermediates are marked. * non-specific bands. (b-d) γ-H2A ChIP-seq enrichment in *ndt80Δ* cultures in early prophase cells (T=3 hrs). Enrichment is plotted along each of the 16 yeast chromosomes in (b), black triangles mark the centromeres and the red hexagon marks the rDNA locus. The data are normalized to a global mean of 1. γ-H2A is enriched around previously defined meiotic chromosome axis summits[1] with the highest enrichment around the strongest summits in (c). γ-H2A is depleted around DSB hotspots[2] in (d). (e) Mean Rad51 enrichment in the EARs (32 domains) is plotted as a function of the distance

from telomeres. (f) Bootstrap-derived distribution within two 90-kb bins per chromosome is shown as a violin plot and the horizontal lines within the plot represent the median and the two-ended 95% CIs. The mean Rad51 ChIP-seq enrichment in EARs (20-110 kb) is shown as an orange/red dot. (g) Time series of S1-seq signal reflecting resected DSB ends[3] from all chromosomes excluding the shortest 3 chromosomes (chromosome I, VI, III) normalized to signal average are plotted as mean signal in EARs (20-110 kb, 26 domains) and interstitial regions (>110 kb from telomeres, 16 domains). The grey dashed line shows the genome average. *** $P < 0.001$ and * $P < 0.05$, ANOVA on mean enrichment followed by a post-hoc Tukey test. (h) Spo11-oligo counts within hotspots[4,5] from all chromosomes excluding the 3 shortest chromosomes (chromosome I, VI, III) normalized to signal average are plotted as mean signal in EARs (20-110 kb, 26 domains) and interstitial regions (>110 kb from either end of all chromosomes, 16 domains). The grey dashed line shows the genome average. ** $P < 0.01$, ANOVA on mean enrichment followed by a post-hoc Tukey test.

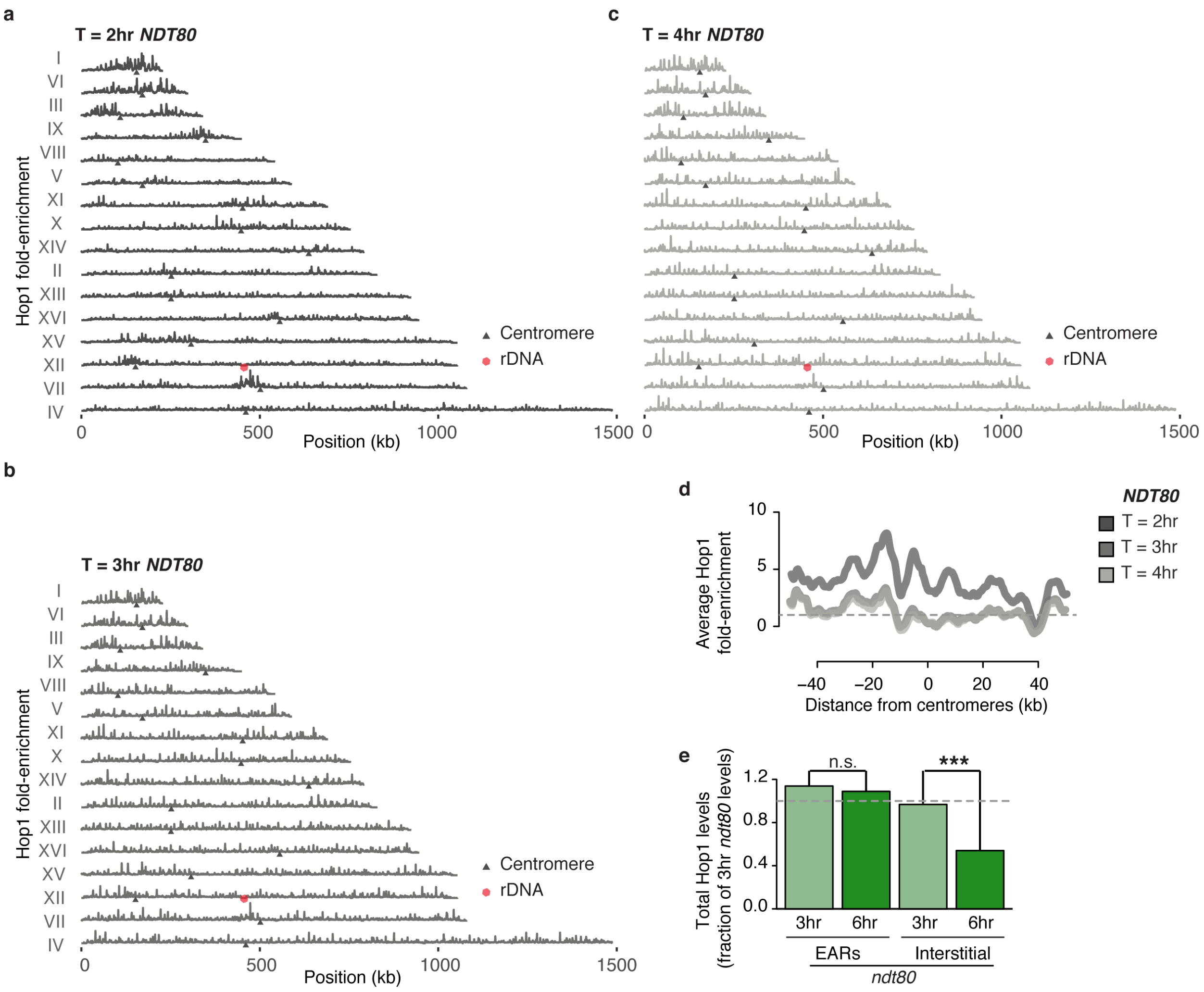

Supplementary Figure 2. Hop1 ChIP-seq enrichment in *NDT80* cultures.

(a-c) Enrichment of Hop1 is plotted along each of the 16 yeast chromosomes at two (a), three (b) and four (c) hours after meiotic induction, black triangles mark the centromeres and the red hexagon marks the rDNA locus. The data are normalized to a global mean of 1. (d) Hop1 enrichment plotted as a mean of the 16 centromeres as function of the distance from centromeres. (e) Spike-in normalized Hop1 levels from early prophase (T=3 hrs) and late prophase (T=6 hrs) *ndt80Δ* samples plotted as mean enrichment in EARs (20-110 kb) and interstitial chromosomal regions. *** $P = 3.33 \times 10^{-9}$ and n.s. $P = 0.29$, Mann-Whitney-Wilcoxon test.

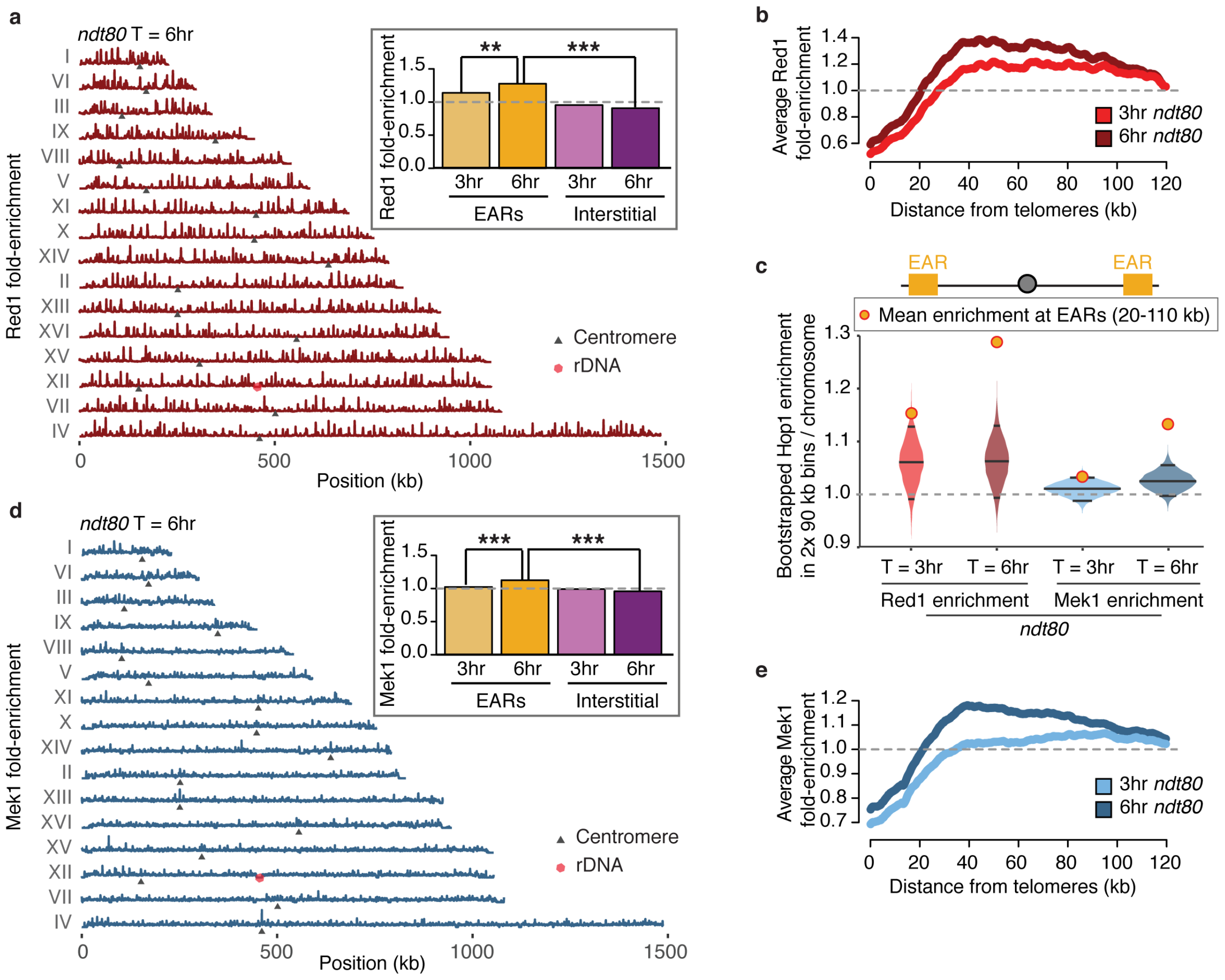

Supplementary Figure 3. Enrichment of Red1 and Mek1 chromosomal proteins in EARs.

(a) Red1 ChIP-seq enrichment (dark red) in *ndt80Δ*-arrested late prophase cells plotted along each of the 16 yeast chromosomes, black triangles mark the centromeres and the red hexagon marks the rDNA locus. The data are normalized to a global mean of 1. Inset shows mean enrichment in EARs (20-110 kb, orange) and interstitial chromosomal regions (magenta) in early prophase (T=3 hrs) and late prophase (T=6 hrs). *** $P < 0.001$ and ** $P < 0.01$, Mann-Whitney-Wilcoxon test. (b) Mean Red1 enrichment in the EARs (32 domains) is plotted as a function of the distance from telomeres. (c) Bootstrap-derived distributions within two 90-kb bins per chromosome from Red1 and Mek1 ChIP-seq are shown as violin plots and the horizontal lines within the plots represent the median and the two-ended 95% CIs. The mean ChIP-seq enrichment in the EARs (20-110 kb) for the respective samples is shown as orange/red dots. (d) Mek1 ChIP-seq enrichment in *ndt80Δ*-arrested late prophase cells plotted along each of the 16 yeast chromosomes, black triangles mark the centromeres and the red hexagon marks the rDNA locus. The data are normalized to a global mean of 1. Inset shows mean enrichment in

EARs (20-110 kb, orange) and interstitial chromosomal regions (magenta) in early prophase (T=3 hrs) and late prophase (T=6 hrs). *** $P < 0.001$ and ** $P < 0.01$, Mann-Whitney-Wilcoxon test. (e) Mean Mek1 enrichment in the EARs (32 domains) is plotted as a function of the distance from telomeres.

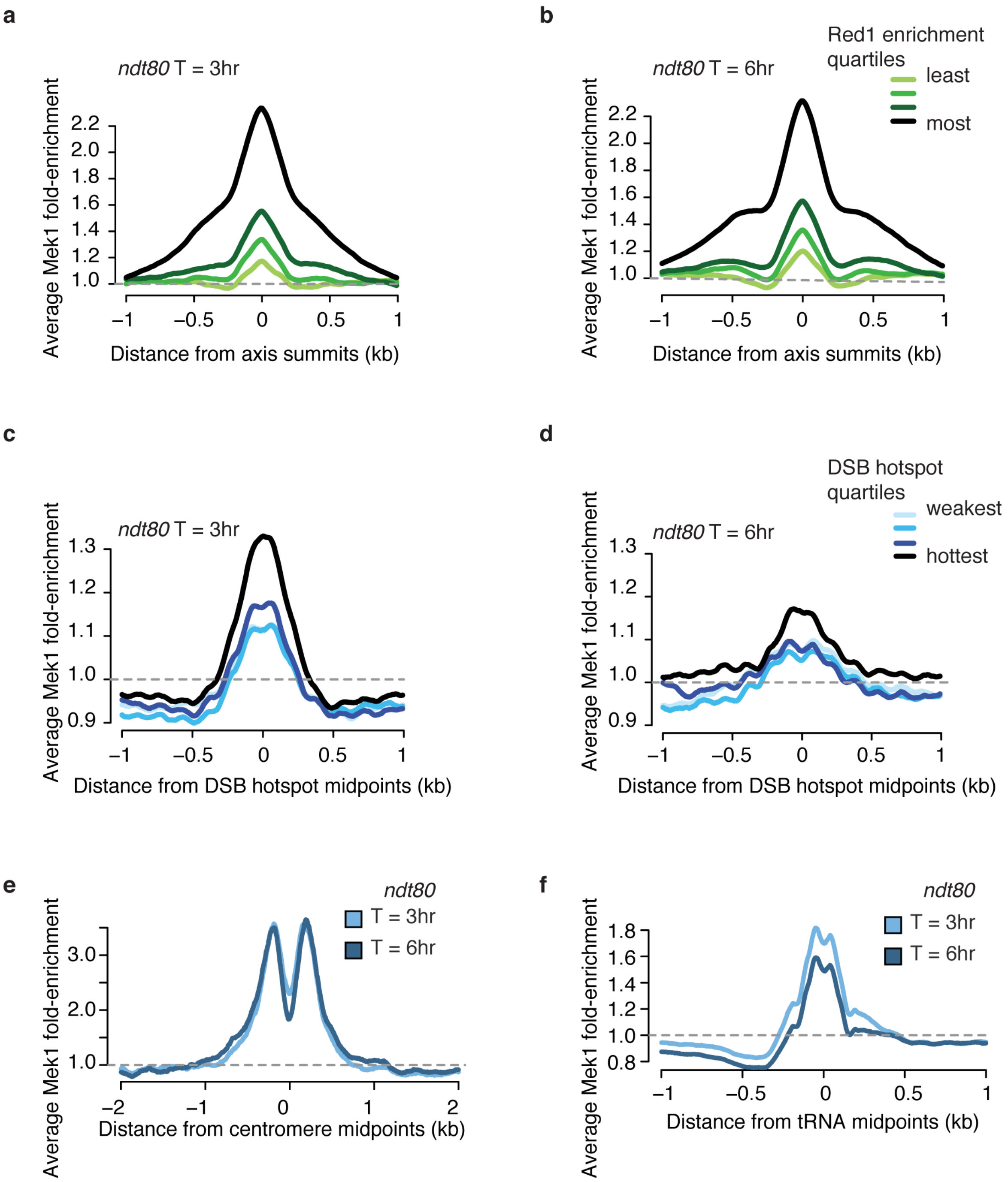

Supplementary Figure 4. Regional Mek1 enrichment during prophase in *ndt80Δ*-arrested cells. (a, b) Mek1 enrichment from early and extended prophase samples around previously defined meiotic axis summits (split in quartiles according to strength of Red1 enrichment; from[1]). (c, d) Mek1 enrichment from early and late prophase samples around previously defined meiotic DSB hotspots (split in quartiles according to strength of DSB hotspots; from[2]). (e) Mek1 enrichment from early and late prophase samples around around centromeres. (f) Mek1 enrichment from early and late prophase samples around around tRNA genes.

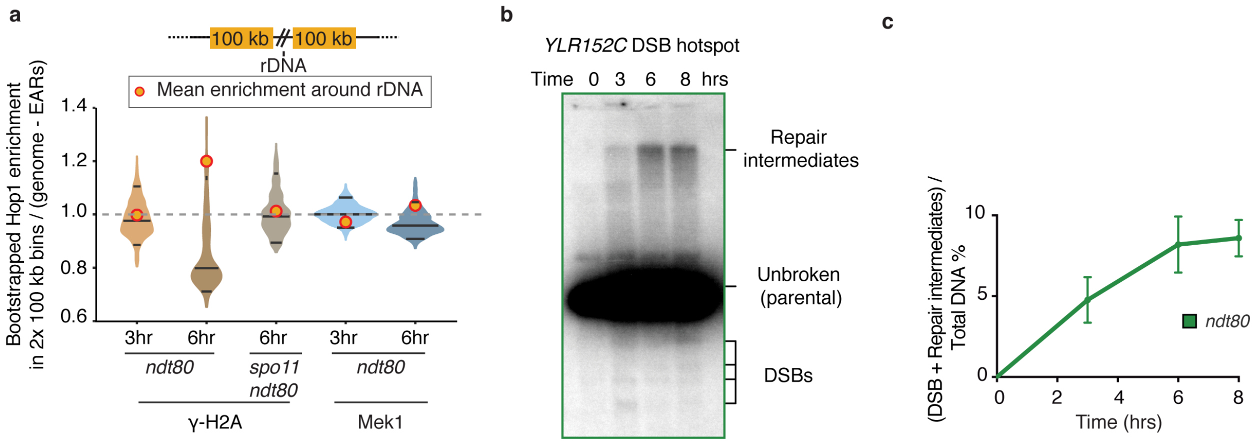

Supplementary Figure 5. Red1 and Mek1 enrichment at rDNA borders.

(a) Bootstrap-derived distributions of γ-H2A and Mek1 ChIP-seq enrichment within two 100-kb bins per genome (lacking EARs) are shown as violin plots and the horizontal lines within the plots represent the median and the two-ended 95% CIs. The mean ChIP-seq enrichment at rDNA borders (100 kb on either side of the rDNA) for the respective samples is shown as orange/red dots. (b) Southern analysis to monitor DSBs and repair intermediates at *YLR152C* DSB hotspot adjacent to the rDNA. (c) The percentage of DSB formation at the *YLR152C* locus over total DNA at the indicated time points. DSBs are plotted as the sum of DSBs and repair intermediates. The data are mean of three independent biological replicates and error bars represent standard deviation from the mean.

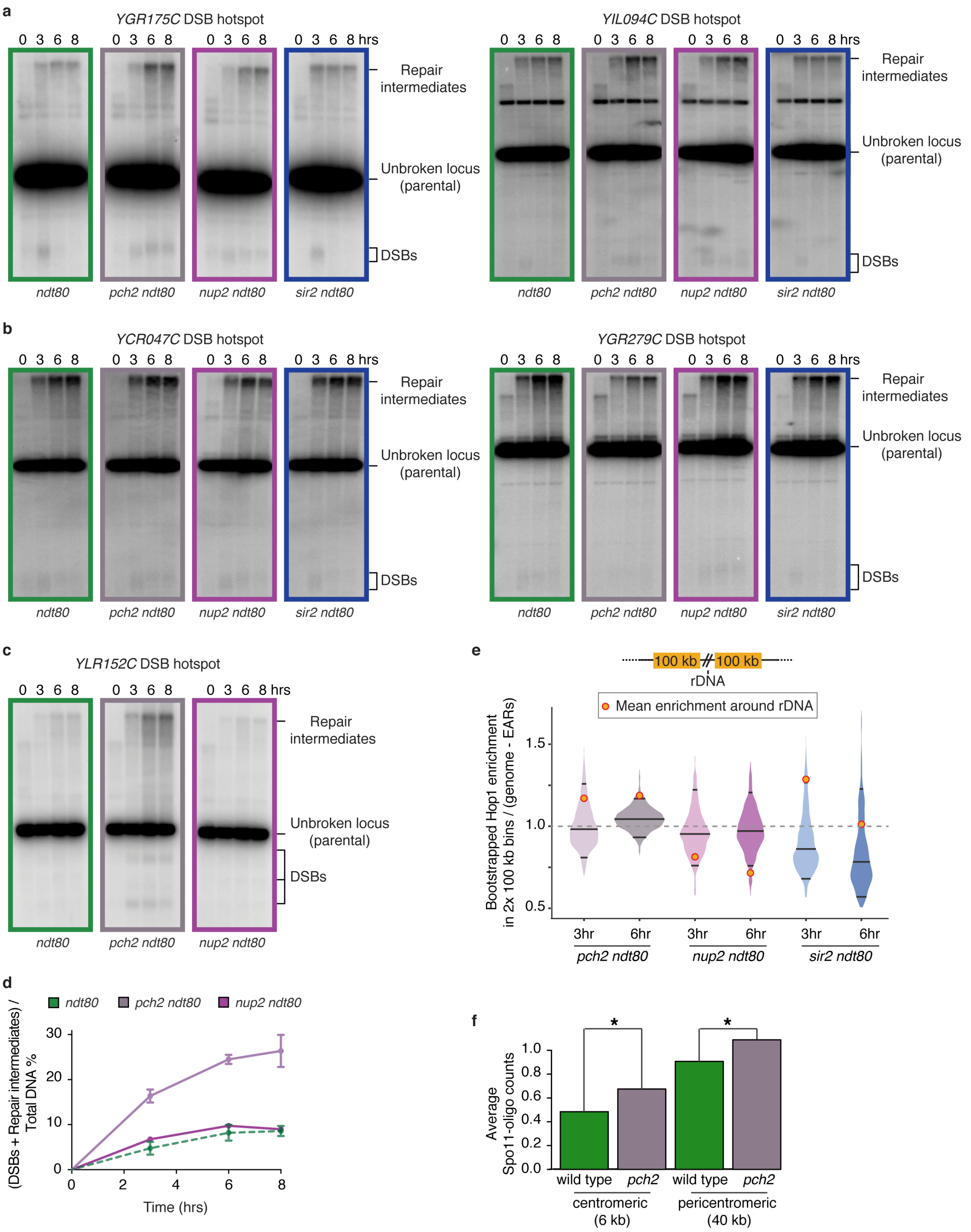

Supplementary Figure 6. Regional Hop1 enrichment and DSB profile.

(a) Southern analysis to monitor DSBs and repair intermediates at the interstitial DSB loci, *YGR175C* and *YIL094C*, in *ndt80Δ*, *pch2Δ ndt80Δ*, *nup2Δ ndt80Δ*, and *sir2Δ ndt80Δ* mutants. (b) Southern analysis to monitor DSBs and repair intermediates at DSB loci in EAR regions,

YCR047C and *YGR279C*, in *ndt80Δ*, *pch2Δ ndt80Δ*, *nup2Δ ndt80Δ*, and *sir2Δ ndt80Δ* mutants. (c) Southern analysis to monitor DSBs and repair intermediates at the *YLR152C* DSB hotspot adjacent to the rDNA in *ndt80Δ*, *pch2Δ ndt80Δ* and *nup2Δ ndt80Δ* mutants. (d) The percentage of DSB formation at the *YLR152C* locus over total DNA at the indicated time points. DSBs are plotted as the sum of DSBs and repair intermediates. The *ndt80Δ* and *pch2Δ ndt80Δ* data are mean of three independent biological replicates and error bars represent standard deviation from the mean. Data for *nup2Δ ndt80Δ* are from a single experiment. The *ndt80Δ* data in green is from Figure S2E. (e) Bootstrap-derived distributions of Hop1 ChIP-seq data within two 100-kb bins per genome (lacking EARs) are illustrated as violin plots. The horizontal lines in the violin plots represent the median and the two-ended 95% CIs. The mean Hop1 ChIP-seq enrichment at rDNA borders (100 kb) for *pch2Δ ndt80Δ*, *nup2Δ ndt80Δ*, and *sir2Δ ndt80Δ* mutants is shown as orange/red dots. (f) Spo11-oligo counts from wild type and *pch2Δ* normalized to signal average are plotted as mean signal around centromeres (6-kb region) and pericentromeres (40-kb region). * $P < 0.05$, Mann-Whitney-Wilcoxon test.

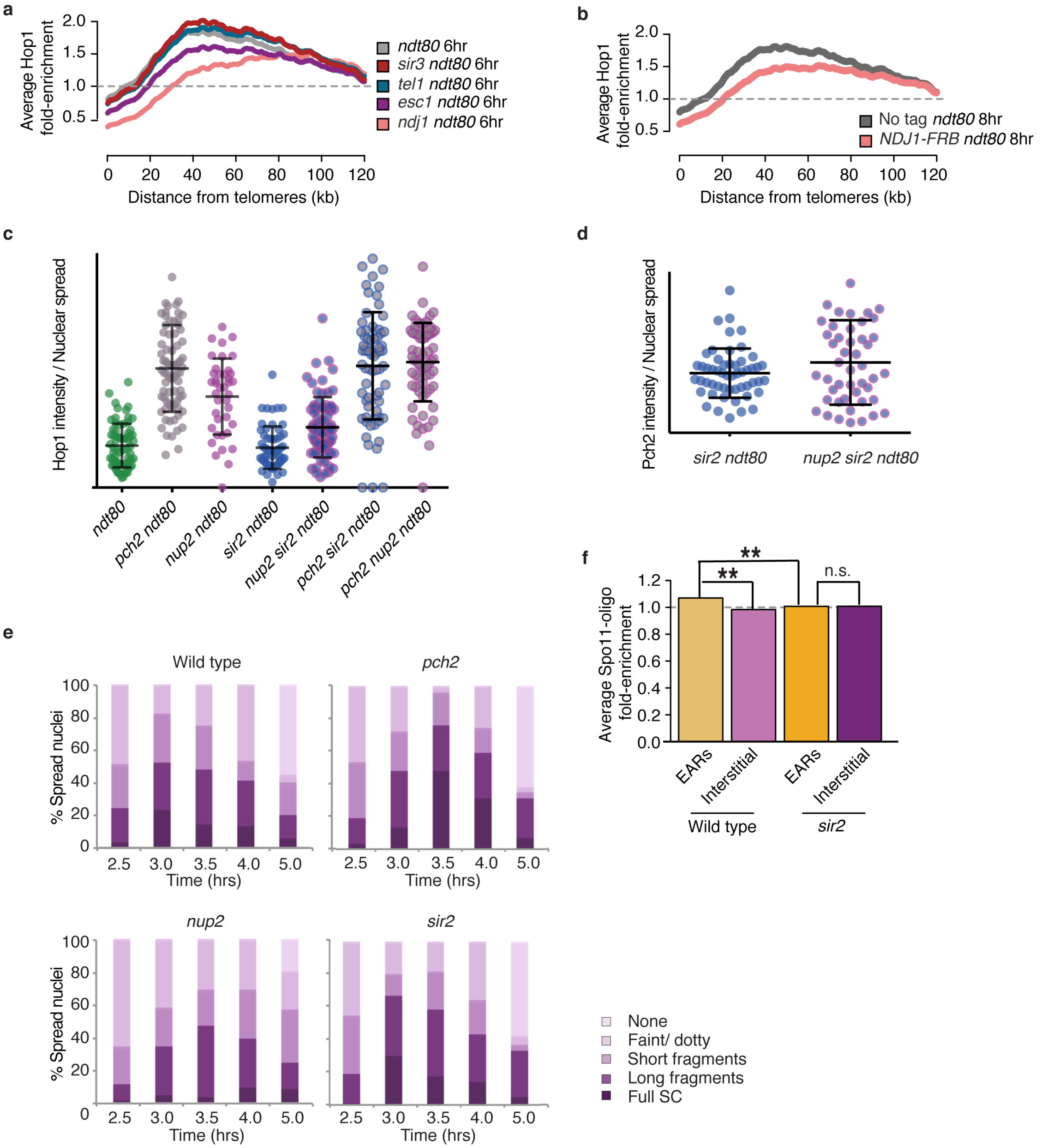

Supplementary Figure 7. Network of proteins regulating Hop1 binding, DSBs and synapsis. (a, b) Mean Hop1 enrichment in the EARs (32 domains) is plotted as a function of the distance from telomeres. Hop1 enrichment data for *ndt80Δ*, *esc1Δ ndt80Δ*, *tel1Δ ndt80Δ*, *sir3Δ ndt80Δ*, and *ndj1Δ ndt80Δ* is shown in (a). The *ndt80Δ* control enrichment in grey is from Figure 2D (T=6 hrs). (b) Hop1 enrichment data at T=8 hrs following 2 hrs of rapamycin treatment in anchor away strains to conditionally deplete Ndj1. Hop1 enrichment in no tag *ndt80Δ* control, and

NDJ1-FRB ndt80Δ are shown. (c) Quantification of total Hop1 intensity per spread nucleus for different mutants. n > 50; error bars are S.D. of the mean. (d) Quantification of total Pch2 intensity per spread nucleus for *sir2Δ ndt80Δ*, and *nup2Δ sir2Δ ndt80Δ* mutants. n > 40; error bars are S.D. of the mean. (e) Quantification of SC assembly progression through meiosis at the indicated time points in wild type, *pch2Δ*, *nup2Δ*, and *sir2Δ* strains (*NDT80*). (f) Spo11-oligo signals from wild type control[5] and *sir2Δ* mutant are normalized to genome average and plotted as mean signal in EARs (20-110 kb, 32 domains) and interstitial chromosomal regions (>110 kb from either end of all chromosomes, 16 domains). The grey dashed line is the genome average. ** $P < 0.01$, n.s. not significant, ANOVA on mean enrichment followed by a post-hoc Tukey test.

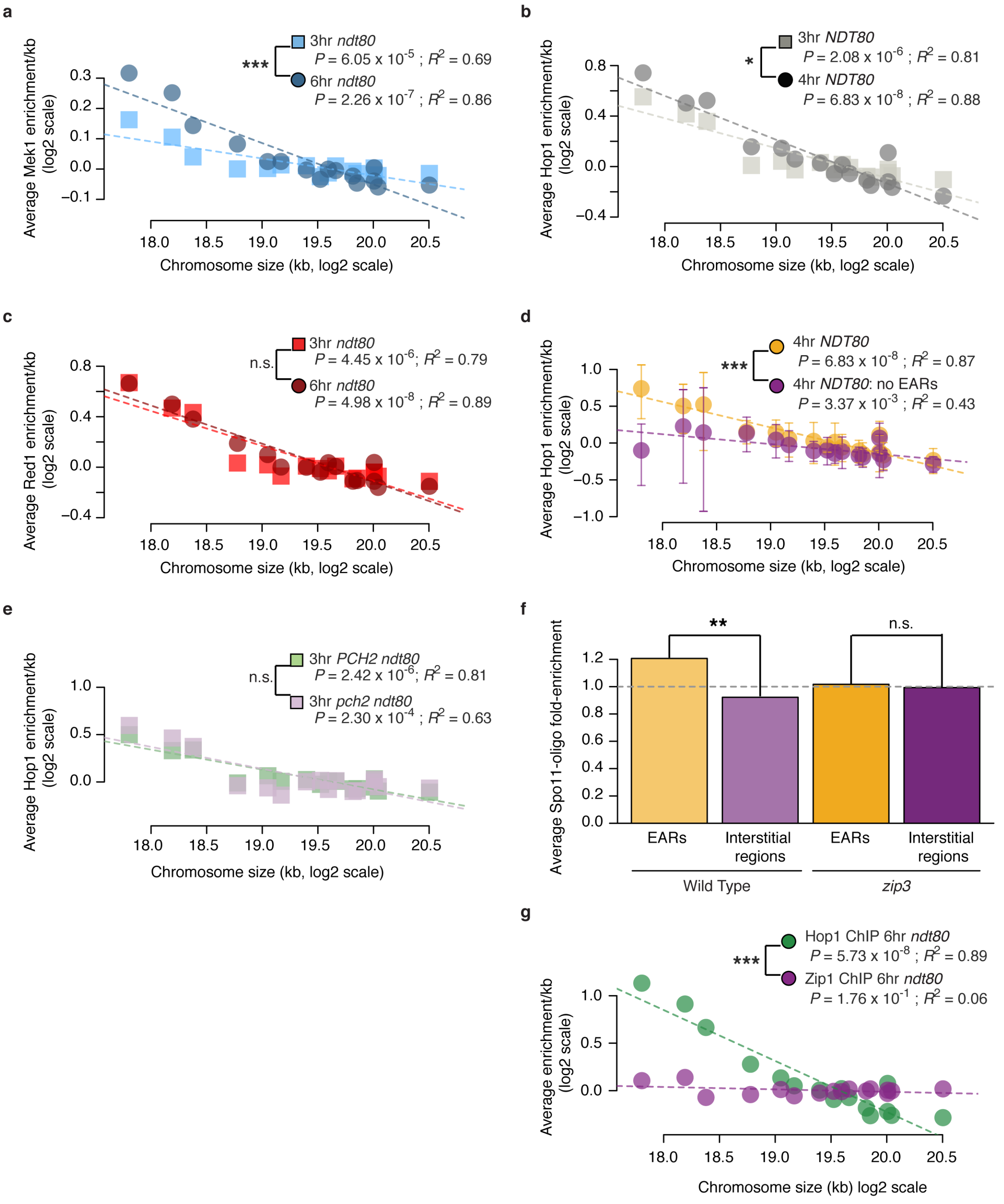

Supplementary Figure 8. Chromosome-size dependent enrichment of proteins in prophase. (a-e) Mean ChIP-seq enrichment per kb is plotted for each chromosome on log scale with regression analysis. *P* and R^2 values are noted below the sample name. R^2, measure of the fit of the points to the line, can vary from 0-1.0 with 1.0 indicating a perfect fit. *P* is the probability of

obtaining large R^2 values. ANOVA was performed to test significant difference in the slope between the regression lines for different ChIP-seq samples. ANOVA-derived *P* values are indicated. Mek1 enrichment in *ndt80Δ* samples at early and late prophase in (a) have significantly different slopes, *** $P < 0.001$. Hop1 enrichment in *NDT80* samples at 3 and 4 hrs in (b) have significantly different slopes, * $P < 0.05$. Red1 enrichment in *ndt80Δ* samples at early and late prophase in (c) do not have significantly different slopes, $P = 0.604$. (d) Hop1 ChIP-seq in late prophase from *NDT80* samples (T=4 hrs) is plotted in orange. Plot in magenta is Hop1 enrichment from telomere-distal regions lacking EARs (110 kb). Error bars are standard deviation of the means of 10 equal sized bins for each chromosome. Hop1 enrichment in *PCH2 ndt80Δ* and *pch2Δ ndt80Δ* samples at early prophase in (e) do not have significantly different slopes, $P = 0.691$, not significant (n.s.). (f) Spo11-oligo counts within hotspots [4] normalized to genome average are plotted as mean signal in EARs (20-110 kb, 32 domains) and interstitial domains (within 110 kb from either end of all chromosomes, 16 domains) from wild-type and *zip3Δ* cultures. ** $P < 0.01$ or not significant (n.s.), Mann-Whitney-Wilcoxon test. (g) Mean Zip1 ChIP-seq enrichment (magenta) and Hop1 ChIP-seq enrichment (green) per kb from *ndt80Δ* samples (T=6 hrs) are plotted for each chromosome on log scale with regression analysis. *P* and R^2 values are noted below the sample name. ANOVA-derived *P* values to test significant difference in the slope between the regression lines are indicated. Zip1 ChIP-seq enrichment (magenta) in *ndt80Δ* samples at late prophase does not exhibit reduction on short chromosomes and is significantly different from Hop1 (green), *** $P < 0.001$.

Supplementary Table 1. Yeast strains utilized in this study.

Strain #	Genotype
AH574	*MATa, ho::LYS2, lys2, LEU2, HIS4, URA3, TRP1*<br>*MATα, ho::LYS2, lys2, leu2::hisG, his4X, URA3, TRP1*
AH6179	*MATa, ho::LYS2, lys2, HIS, URA, LEU, TRP, ndt80Δ::TRP1*<br>*MATα, ho::LYS2, lys2, leu2::hisG, his4X, ura3, trp1::hisG, ndt80Δ::TRP1*
AH6214	*MATa, ho::LYS2, lys2, HIS, URA, LEU, TRP, arg4-Bgl, spo11-Y135F::KanMX4, ndt80Δ::TRP1*<br>*MATα, ho::LYS2, lys2, HIS, ura3::hisG, leu2::hisG, TRP, spo11-Y135F::KanMX4, ndt80Δ::TRP1*
AH6639	*MATa, ho::LYS2, lys2, his4X, URA, leu2::hisG, TRP, pch2Δ::KanMX4, ndt80Δ::TRP1*<br>*MATα, ho::LYS2, lys2, HIS, ura3, LEU, TRP, pch2Δ::KanMX4, ndt80Δ::TRP1*
AH7121	*MATa, ho::LYS2, lys2, URA3, leu2::hisG, his3::hisG, trp1::hisG, ndt80Δ::TRP1, RPL13A-2xFKBP12::TRP1, fpr1Δ::KanmX4, tor1-1::HIS3, RAD54-FRB::KanMX6*<br>*MATα, ho::LYS2, lys2, URA3, LEU2, his3::hisG, trp1::hisG, ndt80Δ::TRP1, RPL13A-2xFKBP12::TRP1, fpr1Δ::KanmX4, tor1-1::HIS3, RAD54-FRB::KanMX6*
AH7137	*MATa, ho::LYS2, lys2, LEU2, ura3, trp1::hisG , his3, RPL13A-2xFKBP12::TRP1, fpr1Δ::KanmX4, tor1-1::HIS3, ndt80Δ::TRP1*<br>*MATα, ho::LYS2, lys2, URA3, LEU2, his3::hisG, trp1::hisG, fpr1Δ::KanmX4, RPL13A-2xFKBP12::TRP1, tor1-1::HIS3, ndt80Δ::TRP1*
AH8912	*MATa, ho::LYS2, lys2, URA3, leu2::hisG, his3::hisG, trp1::hisG, tel1Δ::HIS3, ndt80Δ ::TRP1*<br>*MATα, ho::LYS2, lys2, ura3, LEU2, his3::hisG, trp1::hisG, tel1Δ::HIS3, ndt80Δ ::TRP1*
AH8913	*MATa, lys2, ho::LYS2, TRP1, HIS3, LEU2, URA3, esc1Δ::hphMX4, ndt80Δ::TRP1*<br>*MATα, lys2, ho::LYS2, trp1::hisG(or TRP+), his3::hisG, leu2::hisG, ura3, esc1Δ::hphMX4, ndt80Δ::TRP1*
AH8967	*MATa, ho::LYS2, lys2, ura3, LEU2, his3::hisG, trp1::hisG, sir3Δ::HIS3, ndt80Δ ::TRP1*<br>*MATα, ho::LYS2, lys2, ura3::YIplac211::URA3, LEU2, his3::hisG, trp1::hisG, sir3Δ::HIS3, ndt80Δ ::TRP1*
AH9056	*MATa, ho::LYS2, lys2, his, URA, LEU, TRP, ndt80Δ::TRP1, ndj1Δ::KanMX*<br>*MATα, ho::LYS2, lys2, leu2::hisG, HIS3, HIS4, ura3, trp1::hisG, ndt80Δ::TRP1, ndj1Δ::KanMX*

AH9098	*MATa, ho::LYS2, lys2, LEU2, ura3, trp1::hisG , his3, RPL13A-2xFKBP12::TRP1, NDJ1-FRB::HphMX6, fpr1Δ::KanMX4, tor1-1::HIS3, ndt80Δ ::TRP1*<br>*MATα, ho::LYS2, lys2, URA3, LEU2, his3::hisG, trp1::hisG, NDJ1-FRB::HphMX6, RPL13A-2xFKBP12::TRP1, tor1-1::HIS3, ndt80Δ::TRP1, fpr1Δ::KanMX4*
AH9618	*MATa, lys2, ho::LYS2, trp1::hisG, his3::hisG, leu2::hisG, ura3, CEN1::URA3, CEN2::LEU2, can1, cyh2*<br>*MATα, ho::LYS2, lys2, ura3, leu2::hisG, his3::hisG, trp1::hisG, CEN1::HIS3, CEN2::TRP1, sir2Δ::HPHMX*
AH9697	*MATa, ho::LYS2, lys2, ura3, leu2::hisG, his3::hisG, trp1::hisG, CEN1::HIS3, CEN2::TRP1, pch2Δ::KanMX*<br>*MATα, lys2, ho::LYS2, trp1::hisG, his3::hisG, leu2::hisG, ura3, CEN1::URA3, CEN2::LEU2, pch2Δ::KanMX, can1, cyh2, sir2Δ::hphMX*
AH9740	*MATa, lys2, ho::LYS2, trp1::hisG, his3::hisG, leu2::hisG, ura3, CEN1::HIS3, CEN2::TRP1, sir2Δ::HPHMX*<br>*MATα, ho::LYS2, lys2, ura3, leu2::hisG, his3::hisG, trp1::hisG, CEN1::URA3, CEN2::LEU2, can1, cyh2, sir2Δ::KanMX*
AH9817	*MATa, lys2, ho::LYS2, trp1::hisG, his3::hisG, leu2::hisG, ura3, CEN1::HIS3, CEN2::TRP1, nup2Δ::KanMX4, sir2Δ::HPHMX*<br>*MATα, ho::LYS2, lys2, ura3, leu2::hisG, his3::hisG, trp1::hisG, CEN1::URA, CEN2::LEU2, nup2Δ::KanMX, can1, cyh2*
AH8644	S288c background<br>*MATa, his3Δ1, LEU, LYS, ura3Δ0, RME1(ins-308a), TAO3(E1493Q), MKT1(D30G)*<br><br>*MATα, HIS3, leu2Δ0, lys2Δ0, URA3, RME1(ins-308a), TAO3(E1493Q), MKT1(D30G)*
SKY3821	*MATa; ho::LYS2, lys2, ura3, leu2, arg4-Bgl, nuc1Δ::LEU2, SPO11-His6-flag3-loxP-kanMX-loxP*<br>*MATα; ho::LYS2, lys2, ura3, leu2, arg4-Bgl, nuc1Δ::LEU2, SPO11-His6-flag3-loxP-kanMX-loxP*
SKY4256	*MATa; ho::LYS2, lys2, ura3, leu2, arg4-Bgl, nuc1Δ::LEU2, SPO11-His6-flag3-loxP-kanMX-loxP, sir2Δ::HphMX*<br>*MATα; ho::LYS2, lys2, ura3, leu2, arg4-Bgl, nuc1Δ::LEU2, SPO11-His6-flag3-loxP-kanMX-loxP, sir2Δ::HphMX*
SKY4264	*MATa; ho::LYS2, lys2, ura3, leu2, arg4-Bgl, nuc1Δ::LEU2, SPO11-His6-flag3-loxP-kanMX-loxP, pch2Δ::HphMX*<br>*MATα; ho::LYS2, lys2, ura3, leu2, arg4-Bgl, nuc1Δ::LEU2, SPO11-His6-flag3-loxP-kanMX-loxP, pch2Δ::HphMX*

Supplementary Table 2. Experimental design for DSB Southern analysis.

**DSB locus**	**Restriction enzyme** (Parental size)	**Primer 1 (probe design)**	**Primer 2 (probe design)**
YCR047c	HindIII (5.9 kb)	5' ggaattccgagagaatcgacttgctaa	5' ggaattccagccaccagtgggcttttc
YER004w	BglII (6.1 kb)	5' ctgttgggaaagcaaccaggagaaac	5' ggagactacttttgatgagaaacttggtc
YER024w	SacII/BanII (7.6 kb)	5' cagttttcactctggcctaacg	5' ggatctgtaaaatttcatggcctg
YGR175c	HindIII (6.0 kb)	5' ggcagcaacatatctcaaggcc	5' tcaatgtagcctgagattgtggcg
YGR279c	AseI (7.8 kb)	5' ttcctcgttcgtgacactactc	5' tagctgccaaacccattctgc
YIL094c	AseI (5.2 kb)	5' attgtgcctgtaaccgaactgc	5' agtggacgtagaaagaggagc
YLR152c	HindIII (9.3 kb)	5' agaactccaatgcgcctgggcttg	5' cgtaggcatacgtcgaggaactac
YOL001w	HindIII (9.1 kb)	5' caagttcatcaaaacgtggcc	5' tcccagatagaagggaagcag
YOL081w	BamHI (13.1 kb)	5' ggctaggtctaatggcttagatgtaaga g	5' agccctcgtaaacctcctgtaac

Supplementary Table 3. Antibodies used for ChIP-seq and immunofluorescence experiments

**Antibody**	**Source**	**Quantity used per ChIP**	**Quantity used for immunofluorescence**
Anti-Hop1	Nancy Hollingsworth	2 μL	1:200
Anti-Red1 (Lot# 16440)	Nancy Hollingsworth	2 μL	--
Anti-Mek1	Pedro San-Segundo[6]	2 μL	--
Anti-γ-H2A	Abcam (ab15083)	2 μg	--
Anti-Rad51*	Santa Cruz (SC-33626)	2 μg	--
Anti-Zip1 (yC-19)	Santa Cruz (SC-15632)	2 μg	1:200
Anti-Zip1 (y-300)	Santa Cruz (SC-33733)	2 μg	--
Anti-FLAG	Sigma (F1804)	105 μg	--
Anti-Pch2	Akira Shinohara[7]	--	1:200
Anti-Rabbit⌘	Jackson Laboratory (711-545-152)	--	1:200
Anti-Goat⌘	Jackson Laboratory (705-166-147)	--	1:200

* Rad51 antibody was first pre-adsorbed against *rad51Δ* meiotic spheroplasts.
⌘ Secondary antibodies were pre-adsorbed against yeast spheroplasts.

Supplementary Table 4. ChIP-seq datasets on GEO (GSE105111)

Sample	Time point	Antibody	Sample name
Wild type	2 hr	Anti-Hop1	GSE105111_AH574Hop1T2rep-332-338-Reps-SK1Yue-PM_B3W3_MACS2_FE.bedGraph.gz
Wild type	3 hr	Anti-Hop1	GSE105111_AH574Hop1T3rep-334-340-Reps-SK1Yue-PM_B3W3_MACS2_FE.bedGraph.gz
Wild type	4 hr	Anti-Hop1	GSE105111_AH574Hop1T4rep-336-342-Reps-SK1Yue-PM_B3W3_MACS2_FE.bedGraph.gz
ndt80Δ	3 hr	Anti-Hop1	GSE105111_AH6179Hop1T3rep-118-214-Reps-SK1Yue-PM_B3W3_MACS2_FE.bedGraph.gz
ndt80Δ	6 hr	Anti-Hop1	GSE105111_AH6179Hop1T6rep-121-215-Reps-SK1Yue-PM_B3W3_MACS2_FE.bedGraph.gz
ndt80Δ	3 hr	Anti-Mek1	GSE105111_AH6179Mek1T3rep-117-423-Reps-SK1Yue-PM_B3W3_MACS2_FE.bedGraph.gz
ndt80Δ	6 hr	Anti-Mek1	GSE105111_AH6179Mek1T6rep-120-425-Reps-SK1Yue-PM_B3W3_MACS2_FE.bedGraph.gz
ndt80Δ	3 hr	Anti-Red1	GSE105111_AH6179Red1T3rep-321-428-Reps-SK1Yue-PM_B3W3_MACS2_FE.bedGraph.gz
ndt80Δ	6 hr	Anti-Red1	GSE105111_AH6179Red1T6rep-322-430-Reps-SK1Yue-PM_B3W3_MACS2_FE.bedGraph.gz
ndt80Δ	6 hr	Anti-Zip1	GSE105111_AH6179Zip1T6rep-306-305-Reps-SK1Yue-PM_B3W3_MACS2_FE.bedGraph.gz
ndt80Δ	3 hr	Anti-γ-H2A	GSE105111_AH6179pH2AT3rep-344-348-Reps-SK1Yue-PM_B3W3_MACS2_FE.bedGraph.gz
ndt80Δ	6 hr	Anti-γ-H2A	GSE105111_AH6179pH2AT6rep-346-350-Reps-SK1Yue-PM_B3W3_MACS2_FE.bedGraph.gz
ndt80Δ (+S288c spike)	3 hr	Anti-Hop1	GSE105111_AH6179spikeinHop1T3a_FE.bedGraph.gz
ndt80Δ (+S288c spike)	6 hr	Anti-Hop1	GSE105111_AH6179spikeinHop1T6a_FE.bedGraph.gz

spo11Δ ndt80Δ	2 hr	Anti-γ-H2A	GSE105111_AH6214pH2AT6rep-432-434-Reps-SK1Yue-PM_B3W3_MACS2_FE.bedGraph.gz
pch2Δ ndt80Δ	3 hr	Anti-Hop1	GSE105111_AH6639Hop1T3rep-226-216-Reps-SK1Yue-PM_B3W3_MACS2_FE.bedGraph.gz
pch2Δ ndt80Δ	6 hr	Anti-Hop1	GSE105111_AH6639Hop1T6rep-227-217-Reps-SK1Yue-PM_B3W3_MACS2_FE.bedGraph.gz
pch2Δ ndt80Δ (+S288c spike)	3 hr	Anti-Hop1	GSE105111_AH6639spikeinHop1T3a_FE.bedGraph.gz
pch2Δ ndt80Δ (+S288c spike)	6 hr	Anti-Hop1	GSE105111_AH6639spikeinHop1T3b_FE.bedGraph.gz
pch2Δ ndt80Δ (+S288c spike)	3 hr	Anti-Hop1	GSE105111_AH6639spikeinHop1T6a_FE.bedGraph.gz
pch2Δ ndt80Δ (+S288c spike)	6 hr	Anti-Hop1	GSE105111_AH6639spikeinHop1T6b_FE.bedGraph.gz
ndt80Δ	8 hr	Anti-Rad51	GSE105111_AH7137AH7121Rad51T8rep-360-362-Reps-SK1Yue-PM_B3W3_MACS2_FE.bedGraph.gz
tel1Δ ndt80Δ	6 hr	Anti-Hop1	GSE105111_AH8912T6Hop1a-SK1Yue-PM_B3W3_MACS2_FE.bedGraph.gz
esc1Δ ndt80Δ	6 hr	Anti-Hop1	GSE105111_AH8913Hop1T6rep-315-439-Reps-SK1Yue-PM_B3W3_MACS2_FE.bedGraph.gz
sir3Δ ndt80Δ	6 hr	Anti-Hop1	GSE105111_AH8967T6Hop1a-SK1Yue-PM_B3W3_MACS2_FE.bedGraph.gz
ndt80Δ	8 hr	Anti-Hop1	GSE105111_AH7137T8rapaHop1a-SK1Yue-PM_B3W3_MACS2_FE.bedGraph.gz
ndj1Δ ndt80Δ	6 hr	Anti-Hop1	GSE105111_AH9056T6Hop1a-SK1Yue-PM_B3W3_MACS2_FE.bedGraph.gz
NDJ1-FRB ndt80Δ	8 hr	Anti-Hop1	GSE105111_AH9098T8rapaHop1a-SK1Yue-PM_B3W3_MACS2_FE.bedGraph.gz
sir2Δ ndt80Δ	3 hr	Anti-Hop1	GSE105111_AH9517Hop1T3rep-561-603-Reps-SK1Yue-PM_B3W3_MACS2_FE.bedGraph.gz
sir2Δ ndt80Δ	6 hr	Anti-Hop1	GSE105111_AH9517Hop1T6rep-563-605-Reps-SK1Yue-PM_B3W3_MACS2_FE.bedGraph.gz

nup2Δ ndt80Δ	3 hr	Anti-Hop1	GSE105111_AH9535Hop1T3rep-565-607-Reps-SK1Yue-PM_B3W3_MACS2_FE.bedGraph.gz
nup2Δ ndt80Δ	6 hr	Anti-Hop1	GSE105111_AH9535Hop1T6rep-567-609-Reps-SK1Yue-PM_B3W3_MACS2_FE.bedGraph.gz

Supplementary Table 5. Spo11-oligo datasets on GEO

Sample	Time point	Antibody	GEO - Sample name
Wild type	4 hr	Anti-FLAG	GSE48299 - GSM11174375[4]
Wild type	4 hr	Anti-FLAG	GSE48299 - GSM11174376[4]
zip3Δ	4 hr	Anti-FLAG	GSE48299 - GSM11174377[4]
zip3Δ	4 hr	Anti-FLAG	GSE48299 - GSM11174378[4]
Wild type	4 hr	Anti-FLAG	GSE67910 - GSM1657849[5]
Wild type	4 hr	Anti-FLAG	GSE67910- GSM1657850[5]
pch2Δ	4 hr	Anti-FLAG	GSE122882 - GSM3487638
pch2Δ	4 hr	Anti-FLAG	GSE122882 - GSM3487639
sir2Δ	4 hr	Anti-FLAG	GSE122882 - GSM3487640
sir2Δ	4 hr	Anti-FLAG	GSE122882 - GSM3487641

Supplementary Table 6. S1-seq datasets on GEO (GSE85253)[3]

Sample	Time point	Sample name
Wild type	0 hr	GSM2263204
Wild type	0 hr	GSM2263205
Wild type	2 hr	GSM2263206
Wild type	2 hr	GSM2263207
Wild type	4 hr	GSM2263208
Wild type	4 hr	GSM2263209
Wild type	6 hr	GSM2263210
Wild type	4 hr	GSM2263211

Supplementary References

1. Sun, X. *et al.* Transcription dynamically patterns the meiotic chromosome-axis interface. *Elife* **4**, e07424 (2015).
2. Pan, J. *et al.* A hierarchical combination of factors shapes the genome-wide topography of yeast meiotic recombination initiation. *Cell* **144**, 719-31 (2011).
3. Mimitou, E.P., Yamada, S. & Keeney, S. A global view of meiotic double-strand break end resection. *Science* **355**, 40-45 (2017).
4. Thacker, D., Mohibullah, N., Zhu, X. & Keeney, S. Homologue engagement controls meiotic DNA break number and distribution. *Nature* **510**, 241-6 (2014).
5. Zhu, X. & Keeney, S. High-resolution global analysis of the influences of Bas1 and Ino4 transcription factors on meiotic DNA break distributions in *Saccharomyces cerevisiae*. *Genetics* **201**, 525-42 (2015).
6. Acosta, I., Ontoso, D. & San-Segundo, P.A. The budding yeast polo-like kinase Cdc5 regulates the Ndt80 branch of the meiotic recombination checkpoint pathway. *Mol Biol Cell* **22**, 3478-90 (2011).
7. Subramanian, V.V. *et al.* Chromosome synapsis alleviates Mek1-dependent suppression of meiotic DNA repair. *PLoS Biol* **14**, e1002369 (2016).
