# Supplementary material for: Persistent DNA-break potential near telomeres increases initiation of meiotic recombination on short chromosomes
Source: Nat Commun. 2019 Feb 27;10:970. doi: 10.1038/s41467-019-08875-x (PMC6393486; doi:10.1038/s41467-019-08875-x)
Supplement: Supplementary file 3 — Reporting Summary [file 41467_2019_8875_MOESM3_ESM.pdf]

nature research

Corresponding author(s): Andreas Hochwagen

Reporting Summary

Nature Research wishes to improve the reproducibility of the work that we publish. This form provides structure for consistency and transparency in reporting. For further information on Nature Research policies, see Authors & Referees and the Editorial Policy Checklist.

Statistical parameters

When statistical analyses are reported, confirm that the following items are present in the relevant location (e.g. figure legend, table legend, main text, or Methods section).

n/a	Confirmed	
☐	☒	The exact sample size (*n*) for each experimental group/condition, given as a discrete number and unit of measurement
☐	☒	An indication of whether measurements were taken from distinct samples or whether the same sample was measured repeatedly
☐	☒	The statistical test(s) used AND whether they are one- or two-sided<br>*Only common tests should be described solely by name; describe more complex techniques in the Methods section.*
☒	☐	A description of all covariates tested
☐	☒	A description of any assumptions or corrections, such as tests of normality and adjustment for multiple comparisons
☐	☒	A full description of the statistics including central tendency (e.g. means) or other basic estimates (e.g. regression coefficient) AND variation (e.g. standard deviation) or associated estimates of uncertainty (e.g. confidence intervals)
☐	☒	For null hypothesis testing, the test statistic (e.g. *F*, *t*, *r*) with confidence intervals, effect sizes, degrees of freedom and *P* value noted<br>*Give P values as exact values whenever suitable.*
☒	☐	For Bayesian analysis, information on the choice of priors and Markov chain Monte Carlo settings
☒	☐	For hierarchical and complex designs, identification of the appropriate level for tests and full reporting of outcomes
☐	☒	Estimates of effect sizes (e.g. Cohen's *d*, Pearson's *r*), indicating how they were calculated
☐	☒	Clearly defined error bars<br>*State explicitly what error bars represent (e.g. SD, SE, CI)*

Our web collection on statistics for biologists may be useful.

Software and code

Policy information about availability of computer code

Data collection	Sra-tools version 2.8.1 was used to download previously published data from NCBI's SRA database.
Data analysis	Bowtie version 1.2.0 was used to align sequencing reads to the genomes.<br>Bedtools version 2.25.0 and Samtools version 1.3.1 were used for post-processing and analysis of sequence alignments in SAM and BAM formats.<br>MACS2 version 2.1.1 was used for ChIP-seq data normalization and peak calling.<br>All remaining data analysis was performed using the statistical computing environment R (www.r-project.org). Custom code developed for this study is openly available online at "https://github.com/hochwagenlab/" and "https://github.com/VijiSubramanian/chrEnds"

For manuscripts utilizing custom algorithms or software that are central to the research but not yet described in published literature, software must be made available to editors/reviewers upon request. We strongly encourage code deposition in a community repository (e.g. GitHub). See the Nature Research guidelines for submitting code & software for further information.

Data

Policy information about availability of data

All manuscripts must include a data availability statement. This statement should provide the following information, where applicable:

- Accession codes, unique identifiers, or web links for publicly available datasets
- A list of figures that have associated raw data
- A description of any restrictions on data availability

Data sets have been deposited in NCBI's Gene Expression Omnibus and are accessible through GEO Series accession number GSE105111.

Field-specific reporting

Please select the best fit for your research. If you are not sure, read the appropriate sections before making your selection.

☒ Life sciences ☐ Behavioural & social sciences ☐ Ecological, evolutionary & environmental sciences

For a reference copy of the document with all sections, see nature.com/authors/policies/ReportingSummary-flat.pdf

Life sciences study design

All studies must disclose on these points even when the disclosure is negative.

Sample size	For ChIP-seq analyses, all datasets are averages of 2 biological replicates with exception of some experiments noted in supplementary figure S6AB, which did not show a phenotype and were not repeated. >40 cells were analyzed for all cytological analyses.
Data exclusions	Multiple mappers were removed from Chip-seq samples. For bootstrap analysis of rDNA borders, EAR regions were excluded from the datasets.
Replication	Experiments revealing differences in measured data were repeated at least once and all results were successfully replicated. Quantitative ChIP enrichment for Hop1 was obtained from spike-in normalzed ChIP-seq experiments (SNP-ChIP) which simultaneously measure numerous regions throughout the genome.
Randomization	The study includes no experiments dependent on allocation of samples/organisms/participants into experimental groups.
Blinding	The study includes no experiments dependent on group allocation and blinding is thus not relevant.

Reporting for specific materials, systems and methods

Materials & experimental systems

n/a	Involved in the study
☒	☐ Unique biological materials
☐	☒ Antibodies
☒	☐ Eukaryotic cell lines
☒	☐ Palaeontology
☒	☐ Animals and other organisms
☒	☐ Human research participants

Methods

n/a	Involved in the study
☐	☒ ChIP-seq
☒	☐ Flow cytometry
☒	☐ MRI-based neuroimaging

Antibodies

Antibodies used	Anti-Hop1 (N. Hollingsworth)<br>Anti-Red1 (Lot#16440, N. Hollingsworth)<br>Anti-phospho-H2A-S129 (Abcam #ab15083)<br>Anti-Mek1 (P. San-Segundo)<br>Anti-Rad51 (y-180, Santa Cruz #SC-33626)<br>Anti-Zip1 (yC-19, Santa Cruz #SC-15632)<br>Anti-Zip1 (y-300, Santa Cruz #SC-33733)<br>Anti-FLAG (Sigma #F1804)
Validation	All antibodies have been previously validated by Western blotting using mutant yeast strains affecting the respective epitopes.

ChIP-seq

Data deposition

☒ Confirm that both raw and final processed data have been deposited in a public database such as GEO.

☒ Confirm that you have deposited or provided access to graph files (e.g. BED files) for the called peaks.

Data access links<br>*May remain private before publication.*	Data sets are accessible through GEO Series accession number GSE105111. The record is private until publication. To access for review please use the following link: https://www.ncbi.nlm.nih.gov/geo/query/acc.cgi?acc=GSE105111<br>Enter token 'onmbmkakdfwdxat' into the box
Files in database submission	AH6179A-141013_ATCACG_L003_R1_001.fastq.gz<br>AH6179B-141013_TTAGGC_L003_R1_001.fastq.gz<br>AH6179C-141013_ACTTGA_L003_R1_001.fastq.gz<br>AH6179D-141013_GATCAG_L003_R1_001.fastq.gz<br>AH6179E-141013_TAGCTT_L003_R1_001.fastq.gz<br>AH6179F-141013_GGCTAC_L003_R1_001.fastq.gz<br>C8CR7ACXX_l06n01_vs6179-t3hop1-160326.35100000057890.fastq.gz<br>C8CR7ACXX_l06n01_vs6179-t3input-160326.351000000579fc.fastq.gz<br>C8CR7ACXX_l06n01_vs6179-t6hop1-160326.3510000005775e.fastq.gz<br>C8CR7ACXX_l06n01_vs6179-t6input-160326.35100000057a74.fastq.gz<br>C8CR7ACXX_l06n01_vs6639-t3hop1-160326.35100000057791.fastq.gz<br>C8CR7ACXX_l06n01_vs6639-t3input-160326.35100000057afb.fastq.gz<br>C8CR7ACXX_l06n01_vs6639-t6hop1-160326.351000000577fe.fastq.gz<br>C8CR7ACXX_l06n01_vs6639-t6input-160326.35100000057b73.fastq.gz<br>C8CR7ACXX_l07n01_vs6639-t3hop1-150818.35100000057784.fastq.gz<br>C8CR7ACXX_l07n01_vs6639-t3input-150818.351000000578ad.fastq.gz<br>C8CR7ACXX_l07n01_vs6639-t6hop1-150818.351000000577ba.fastq.gz<br>C8CR7ACXX_l07n01_vs6639-t6input-150818.351000000578fd.fastq.gz<br>H7V7CAFXX_n01_vs29_170203.fastq.gz<br>H7V7CAFXX_n01_vs30_170203.fastq.gz<br>H7V7CAFXX_n01_vs31_170203.fastq.gz<br>H7V7CAFXX_n01_vs32_170203.fastq.gz<br>HFKCHAFXX_n01_vs1_170124.fastq.gz<br>HFKCHAFXX_n01_vs2_170124.fastq.gz<br>HFKCHAFXX_n01_vs4_170124.fastq.gz<br>HFKCHAFXX_n01_vs5_170124.fastq.gz<br>HFKCHAFXX_n01_vs6_170124.fastq.gz<br>HFKCHAFXX_n01_vs7_170124.fastq.gz<br>HFKCHAFXX_n01_vs8_170124.fastq.gz<br>HFKCHAFXX_n01_vs9_170124.fastq.gz<br>HFKCHAFXX_n01_vs10_170124.fastq.gz<br>HFKCHAFXX_n01_vs11_170124.fastq.gz<br>HFKCHAFXX_n01_vs12_170124.fastq.gz<br>HFKCHAFXX_n01_vs13_170124.fastq.gz<br>HFKCHAFXX_n01_vs14_170124.fastq.gz<br>HFKCHAFXX_n01_vs15_170124.fastq.gz<br>HFKCHAFXX_n01_vs16_170124.fastq.gz<br>HFKCHAFXX_n01_vs17_170124.fastq.gz<br>HFKCHAFXX_n01_vs18_170124.fastq.gz<br>HFKCHAFXX_n01_vs19_170124.fastq.gz<br>HFKCHAFXX_n01_vs20_170124.fastq.gz<br>HFKCHAFXX_n01_vs3_170124.fastq.gz<br>HJKYNBCXX_l01n01_vs25_160909.351000000689de.fastq.gz<br>HJKYNBCXX_l01n01_vs26_160909.351000000689f8.fastq.gz<br>HJKYNBCXX_l01n01_vs27_160909.35100000068a13.fastq.gz<br>HJKYNBCXX_l02n01_vs5_160909.35100000068af7.fastq.gz<br>HJKYNBCXX_l02n01_vs9_160909.3510000006898e.fastq.gz<br>HJKYNBCXX_l02n01_vs17_160909.35100000068b12.fastq.gz<br>HJKYNBCXX_l02n01_vs21_160909.35100000068a8d.fastq.gz<br>HJYMHAFXX_n01_vs1_170421.fastq.gz<br>HJYMHAFXX_n01_vs2_170421.fastq.gz<br>HJYMHAFXX_n01_vs3_170421.fastq.gz<br>HJYMHAFXX_n01_vs4_170421.fastq.gz<br>HJYMHAFXX_n01_vs6_170421.fastq.gz<br>HJYMHAFXX_n01_vs7_170421.fastq.gz<br>HJYMHAFXX_n01_vs8_170421.fastq.gz<br>HJYMHAFXX_n01_vs9_170421.fastq.gz<br>HJYMHAFXX_n01_vs10_170421.fastq.gz<br>HJYMHAFXX_n01_vs11_170421.fastq.gz<br>HJYMHAFXX_n01_vs12_170421.fastq.gz<br>HJYMHAFXX_n01_vs13_170421.fastq.gz<br>HVH7LAFXX_n01_vs17_171115.fastq.gz

HVH7LAFXX_n01_vs18_171115.fastq.gz
HVH7LAFXX_n01_vs19_171115.fastq.gz
HVH7LAFXX_n01_vs20_171115.fastq.gz
HW22FAFXX_n01_vs5_171213.fastq.gz
HW22FAFXX_n01_vs6_171213.fastq.gz
HW22FAFXX_n01_vs7_171213.fastq.gz
HW22FAFXX_n01_vs8_171213.fastq.gz
HVH7LAFXX_n01_vs13_171115.fastq.gz
HVH7LAFXX_n01_vs14_171115.fastq.gz
HVH7LAFXX_n01_vs15_171115.fastq.gz
HVH7LAFXX_n01_vs16_171115.fastq.gz
HW22FAFXX_n01_vs1_171213.fastq.gz
HW22FAFXX_n01_vs2_171213.fastq.gz
HW22FAFXX_n01_vs3_171213.fastq.gz
HW22FAFXX_n01_vs4_171213.fastq.gz
HJKYNBCXX_l02n01_vs23_160909.35100000068ab3.fastq.gz
HJKYNBCXX_l02n01_vs19_160909.35100000068a4a.fastq.gz
HJYMHAFXX_n01_vs17_170421.fastq.gz
HJYMHAFXX_n01_vs18_170421.fastq.gz
HVH7LAFXX_n01_vs11_171115.fastq.gz
HVH7LAFXX_n01_vs12_171115.fastq.gz
HJKYNBCXX_l02n01_vs22_160909.35100000068a9a.fastq.gz
HJKYNBCXX_l02n01_vs18_160909.35100000068a20.fastq.gz
HJKYNBCXX_l02n01_vs24_160909.35100000068add.fastq.gz
HJKYNBCXX_l02n01_vs20_160909.35100000068a63.fastq.gz
H7V7CAFXX_n01_vs33_170203.fastq.gz
H7V7CAFXX_n01_vs35_170203.fastq.gz
H7V7CAFXX_n01_vs36_170203.fastq.gz
HW373AFXX_n01_vs29_171218.fastq.gz
HW373AFXX_n01_vs30_171218.fastq.gz
HW373AFXX_n01_vs31_171218.fastq.gz
HW373AFXX_n01_vs32_171218.fastq.gz
H5FFNAFXY_n01_VS5_181029.fastq.gz
H5FFNAFXY_n01_VS6_181029.fastq.gz
H5FFNAFXY_n01_VS7_181029.fastq.gz
H5FFNAFXY_n01_VS8_181029.fastq.gz
H5FFNAFXY_n01_VS9_181029.fastq.gz
H5FFNAFXY_n01_VS10_181029.fastq.gz
H5FFNAFXY_n01_VS11_181029.fastq.gz
H5FFNAFXY_n01_VS12_181029.fastq.gz

Genome browser session (e.g. UCSC)

Not applicable.

Methodology

Replicates

Results pointing to differences between samples were repeated at least once and always showed excellent agreement. All individual data points are explicitly included in the figures.

Sequencing depth

We used 50-bp, single-end reads and sequencing depth was chosen according to previous results and our lab's extensive experience with yeast ChIP-seq experiments.

Antibodies

Anti-Hop1 (N. Hollingsworth)
Anti-Red1 (Lot#16440, N. Hollingsworth)
Anti-phospho-H2A-S129 (Abcam #ab15083)
Anti-Mek1 (P. San-Segundo)
Anti-Rad51 (y-180, Santa Cruz #SC-33626)
Anti-Zip1 (yC-19, Santa Cruz #SC-15632)
Anti-Zip1 (y-300, Santa Cruz #SC-33733)

Peak calling parameters

Peak calling was performed using MACS2 version 2.1.1 with the following parameters:
macs2 callpeak --keep-dup="auto" -B --nomodel --extsize 200 --SPMR -g 2.4e7

Data quality

Data quality of all sequencing runs were confirmed by FastQC. Newly obtained data are all consistent with previous data. Peaks are not a focus of the work.

Software

Same as "Software and code" section here above.
